# The value of multiple diffusion metrics based on whole-lesion histogram analysis in evaluating the subtypes and proliferation status of non-small cell lung cancer

**DOI:** 10.3389/fonc.2024.1434326

**Published:** 2024-10-30

**Authors:** Yao Chen, Hong Yang, Yuan Qin, Chuanjiang Guan, Wenbing Zeng, Yong Luo

**Affiliations:** ^1^ Department of Radiology, Chongqing University Three Gorges Hospital, Chongqing, China; ^2^ Chongqing University School of Medicine, Chongqing, China

**Keywords:** intravoxel incoherent motion, diffusion kurtosis imaging, histogram analysis, non-small cell lung cancer, Ki-67

## Abstract

**Objective:**

Limited studies have explored the utility of whole-lesion histogram analysis in discerning the subtypes and proliferation status of non-small cell lung cancer (NSCLC), despite its potential to provide comprehensive tissue assessment through the computation of additional quantitative metrics. This study sought to assess the significance of intravoxel incoherent motion (IVIM) and diffusion kurtosis imaging (DKI) histogram parameters in discriminating between squamous cell carcinoma (SCC) and adenocarcinoma (AC), and to examine the correlation of each parameter with the proliferative marker Ki-67.

**Materials and methods:**

Patients with space-occupying lesions detected by chest CT examination and with further routine MRI, DKI and IVIM functional sequence scans were enrolled. Based on the pathological results, seventy patients with NSCLC were selected and divided into AC and SCC groups. Histogram parameters of IVIM (D, D*, f) and DKI (D_app_, K_app_) were calculated, and the Mann–Whitney U test or independent samples t test was used to analyze the differences in each histogram parameter of the SCC and AC groups. Receiver operating characteristic (ROC) curves were used to evaluate the diagnostic performance of the histogram parameters. The correlation coefficient between histogram parameters and Ki-67 was calculated using Spearman’s or Pearson’s methods.

**Results:**

The D ^10th percentile^, D ^90th percentile^, D ^mean^, D ^median^, D_app_^10th percentile^, D_app_^90th percentile^, D_app_^mean^, D_app_^median^, D_app_^skewness^, D_app_^SD^ of the AC groups were significantly higher than those of the SCC groups, while the K_app_^entropy^ and K_app_^SD^ of the SCC groups were significantly higher than those of the AC groups. All the above differences were statistically significant (all P < 0.05). ROC curve analysis revealed that D_app_^mean^ showed the best performance for differentiating AC from SCC lesions, with an area under the ROC curve of 0.832 (95% confidence interval [CI]: 0.707-0.919). But there was no statistically significant difference in diagnostic efficacy compared to other histogram parameters (all P>0.05). D_app_^90thpercentile^, D_app_^mean^, K_app_^skewnes^ showed a slight negative correlation with Ki-67 expression (r value -0.340, -0.287, -0.344, respectively; P< 0.05), while the other histogram parameters showed no significant correlation with Ki-67 (all P > 0.05).

**Conclusions:**

Our study demonstrates the utility of IVIM and DKI histogram analyses in differentiating NSCLC subtypes, particularly AC and SCC. Correlations with the Ki-67 index suggest that D_app_^mean^, D_app_^90th percentile^, and K_app_^skewness^ may serve as markers of tumor aggressiveness, supporting their use in NSCLC diagnosis and treatment planning.

## 1 Introduction

Lung cancer remains a leading cause of morbidity and mortality worldwide. In 2020 alone, there were an estimated 2.2 million new cases and 1.8 million deaths attributed to lung cancer globally (1).

It is the most common cause of cancer-related death among men and ranks as the second leading cause of cancer mortality among women, following breast cancer (2). Lung cancer is broadly categorized into two major types: non-small cell lung cancer (NSCLC) and small cell lung cancer (SCLC), with NSCLC accounting for approximately 85% of all cases (3). Within NSCLC, the most prevalent histological subtypes are squamous cell carcinoma (SCC) and adenocarcinoma (AC) (4).

Accurate information regarding the histologic subtype, tumor differentiation degree, and molecular markers of NSCLC is crucial for selecting clinical treatments and evaluating prognosis.

For example, pemetrexed and bevacizumab are more effective for AC, while bevacizumab for SCC carries a risk of pulmonary hemorrhage (5). Meanwhile, the stage-specific 5-year overall survival rates of patients with AC are superior to those of SCC (6). Additionally, immunohistochemical analysis of NSCLC can enhance the prognostic evaluation value of the lung cancer staging system by identifying markers of tumor aggressiveness. Among these markers, Ki-67, as a nuclear antigen associated with cell proliferation, could reflect the proportion of active cells in the cell cycle. Overexpression of Ki-67 showed a significant positive relationship with poorer differentiation, larger tumor volume, and higher pathological stage (7). Moreover, high expression of Ki-67 had negative effects on disease-free survival, recurrence-free survival, and overall survival of NSCLC patients (8).

Although pathological examination remains the gold standard for obtaining histological features of lung cancer, it relies on CT-guided needle biopsy. However, these techniques have shortcomings, including the risk of anesthesia, significant bleeding, potential for pneumothorax, postoperative tumor dissemination, or challenges in wound infection healing. Additionally, some patients may not be suitable candidates for needle biopsy due to compromised lung function, a lesion’s rich blood supply, or its proximity to major blood vessels (9). Moreover, needle biopsy only samples a portion of the tumor.

With the continuous upgrading of magnetic resonance imaging (MRI) technology, it has the advantages of no radiation damage and multisequence and multiparameter imaging and has been gradually applied to the diagnosis of lung diseases (10). Compared to traditional MRI, functional magnetic resonance imaging (fMRI) sequences can obtain anatomical structure and multiple functional information of tissues. Among them, diffusion-weighted imaging (DWI) shows a potential diagnostic value for lung lesions by measuring the diffusion of water molecules in tissues. However, ADC values are influenced by tissue microcirculation perfusion. Compared with traditional DWI, intravoxel incoherent motion MR imaging (IVIM) allows for independent calculation of diffusion and perfusion parameters based on a dual exponential model. From IVIM, three parameters can be obtained: true diffusion coefficient (D value), perfusion-related pseudo diffusion coefficient (D* value), and perfusion fraction (f value). Furthermore, quantitative information on intra lesion perfusion and diffusion can be provided, making up for the shortcomings of traditional single exponential models DWI (11). In addition, DWI assumes that the behavior of water diffusion is Gaussian, which is unlikely to occur in microstructure complex tissues. Therefore, a diffusion kurtosis imaging (DKI) model based on a non-Gaussian distribution has been proposed, which can better reflect the water diffusion rate in ultrahigh b-value tissues (12). Its main parameters include apparent diffusion kurtosis MK (K_app_) and diffusion coefficient MD (D_app_) after kurtosis correction.

Due to these advantages, DKI and IVIM have been used to differentiate benign and malignant pulmonary lesions (12–14). In recent years, only a few studies have explored the value of DKI and IVIM in the assessment of lung cancer subtype and proliferative status, respectively (15–17). However, combined research on NSCLC is lacking. Considering that the IVIM and DKI models provide information on different aspects of tissue microstructure, it is necessary to compare their value in NSCLC and screen out the optimal diagnostic parameters.

In addition, NSCLC is highly heterogeneous regarding genetic and phenotypic features (18), and this heterogeneity can lead to drug resistance and treatment failure in tumors. It’s worth noting that previous studies were limited to single-section regions of interest (ROI) rather than whole-lesion volumes. Traditional ROI analysis cannot adequately capture intratumor heterogeneity and may introduce subjective bias and possible measurement sampling errors. Whole-lesion histogram analysis, on the other hand, can offer comprehensive microstructural information of lesions, including standard deviation, percentile, entropy, skewness, and kurtosis values (19). This volumetric analysis can address internal lesion heterogeneity and has shown better interobserver reproducibility (20). It has been increasingly applied in the differentiation, staging, grading, efficacy, and prognosis evaluation of tumors (21–23).

Therefore, building upon previous studies, this research aims to explore the value of DKI and IVIM histogram parameters in distinguishing SCC and AC, and to assess the correlation of each parameter with the proliferative status Ki-67. This endeavor seeks to provide a new reference for the diagnosis and treatment of NSCLC.

## 2 Materials and methods

### 2.1 Study population

The Medical Ethics Committee of our hospital approved the prospective study protocol. After obtaining written informed consent from each patient, from May 2022 to February 2024, a total of 83 patients with a space-occupying lesion detected by chest CT were enrolled. Subsequently, routine MRI, DKI, and IVIM functional sequence scanning were conducted. Exclusion criteria were applied as follows: (1) solid or partially solid SPL (solid component > 50%) with a diameter ≥15 mm; (2) no recent history of acute inflammation; and (3) MRI scan performed within 1 week after lung lesions were detected by CT examination, with no radiotherapy, chemotherapy, or other treatment performed before the scan. Additional exclusion criteria comprised: (1) MRI contraindications, such as claustrophobia, foreign metal bodies in the body, and inability to complete the examination; (2) parameter values not measured due to serious image artifacts (4 cases were excluded); (3) benign lesions, metastases and other rare tumors (3 cases were excluded); and (4) incomplete pathological results (6 cases were excluded). Pathological results were mainly obtained through surgical resection, CT-guided percutaneous biopsy, or fiberoptic bronchoscopy biopsy, including the tissue type of the pathological lesion and the immunohistochemical parameter Ki-67. Ultimately, a total of 70 patients were included in our study, consisting of 45 males and 25 females, with an age of 61.01 ± 7.32 (range, 46-79) years.

### 2.2 Image acquisition

Within one week after the lesions were detected by CT, all participants underwent scanning with a 3.0T MRI imaging device (MAGNETOM Vida, Siemens Healthcare, Germany) and a 16-channel phased-array coil. The patients were scanned in the supine position while breathing calmly and uniformly, and a STAR-VIBE sequence scan was performed. Initially, conventional axial T1- weighted and axial T2-weighted imaging sequences were acquired. Subsequently, IVIM and DKI images were obtained using multiple b-values. Detailed MR imaging parameters are provided in **Table 1**.

**Table 1:** MR imaging parameters.

Parameter	Axial T1WI	Axial T2WI	Axial IVM	Axial DKI
Imagin technique	3D VIBE	BLADE	SE-EPI	SE-EPI
Respiratory compensation	Breath holding	Respiratory- triggered	Free breathing	Free breathing
TR/TE (ms)	2.41/1.28	4000/95	7000/58	7000/58
FOV(mm²)	380×100	400×100	380×304	380×304
Matrix	320×50	320×100	120×100	120×100
Thickness (mm)	4	4	4	4
Section gap(mm)	0.8	0.8	0.8	0.8
b-values (s/mm²)	/	/	0, 20, 50,100,150, 200, 300, 500, 800, 1000, 1200, 1600, 2000	0,500,1000,1600

### 2.3 Image analysis

The original images of all patients were transferred to Whole Body Diffusion Toolbox software (Siemens Medical Systems) for postprocessing analysis. Multiple b-value DWI data were postprocessed using different models. IVIM parameters [true molecular diffusion coefficient (D), pseudodiffusion coefficient (D*), and perfusion fraction (f)] were obtained with a biexponential model using 13 b-values (s/mm2), where D* represents the incoherent movement of microcirculation in voxels; D represents pure molecular diffusion; and f represents the ratio of the microcirculation perfusion-related diffusion effect in voxels to the total diffusion effect. Subsequently, the DKI model was selected for functional calculation using 4 b-values (b = s/mm²). DKI parameters [apparent diffusional kurtosis (K_app_) and kurtosis-corrected diffusion coefficient (D_app_)] were derived, where K_app_ represents the deviation of water motion from Gaussian diffusion and reflects the heterogeneity and complexity of tissue microstructure, while D_app_ is the kurtosis-corrected diffusion coefficient (13).

The whole-lesion ROI was manually delineated layer by layer on ADC maps and avoided visible liquefaction necrosis, cavities, bronchi, large blood vessels and the marginal area of the tumor by referring to T2WI and T1WI images because these areas have no tumor cell activity, which will affect the analysis results. The delineated ROIs were fused into a volume of interest (VOI) and then copied to other functional imaging images. The corresponding histogram parameters of IVIM(D, D* and f) and DKI (K_app_, D_app_) were obtained:(1) 10th percentile, (2) 90th percentile, (3) mean, (4) median, (5) entropy, (6) kurtosis, (7) skewness, (8) uniformity and (9) standard deviation (SD). All the quantitative parameters described above were independently processed and measured by two experienced radiologists who were blinded to the clinical or pathological information.

### 2.4 Pathological analysis

All tumor specimens obtained were sent to the Department of Pathology of our hospital for fixation, dehydration, wax immersion, embedding, sectioning and routine HE staining and immunohistochemical staining, recording the results of tissue specimen pathology type and immunohistochemical staining. Pathological classification was based on the 2021 World Health Organization classification criteria for lung, pleura, thymus, and cardiac tumors (24). The expression of Ki-67 was analyzed by immunohistochemistry.

### 2.5 Statistical analysis

SPSS 25.0, MedCalc 19.4, and GraphPad Prism 9.0 software were used for statistical analysis and graph drawing. The intraclass correlation coefficient (ICC) was used to evaluate the consistency of each parameter by two radiologists (r ≥ 0.75, excellent agreement, 0.60 ≤ r<0.75, good agreement, 0.40 ≤ r< 0.60, fair agreement; and r< 0.40, poor agreement) (25). The Shapiro-Wilk test was used to test the normality of the measurement data. Quantitative data were expressed as the mean ± standard deviation (SD) or median and interquartile ranges based on the distribution. Measurement data with a normal distribution were analyzed by independent samples t tests; otherwise, the Mann–Whitney U test was used to compare the differences in each parameter between the AC and SCC groups. Enumeration data were described by the number of cases (percentage), and comparisons between groups were performed by the X^2^ test or Fisher’s exact probability method. The ROC curves were used to evaluate the diagnostic efficacy of each histogram parameter in differentiating AC and SCC groups. The areas under the curve (AUCs), sensitivity, specificity, and Youden index were calculated to determine the optimal diagnostic threshold. The DeLong method was used to compare AUC differences between each parameter. The correlation between each histogram parameter and the Ki- 67 index was assessed in the AC and SCC groups. Pearson correlation analysis was utilized for data with a normal distribution; otherwise, Spearman correlation analysis was employed. A two-tailed P value < 0.05 was considered statistically significant.

## 3 Results

### 3.1 Patient characteristics

A total of 83 patients were recruited for our study, and 13 patients were excluded. The reasons for exclusion were as follows: serious image artifacts (n=4), other tumors or benign lesions (n=3) and the absence of complete pathological results (n=6). Ultimately, a total of 70 patients with NSCLC were included in our study. There were 45 males and 25 females, with an age of 61.01 ± 7.32 (range, 46-79) years. The lesions were confirmed as AC (n=51) or SCC (n=19). The clinical and imaging data are shown in **Table 2**. There were statistically significant differences in sex, smoking history, and Ki-67 between the AC and SCC groups. The analysis revealed that the SCC groups were predominantly male and had more smoking patients, as well as higher Ki-67 values compared to the AC groups. There were no significant differences in age or tumor location.

**Table 2:** Comparison of clinical and imaging data between AC and SCC lesions.

Parameters	AC(n=51)	SCC (n= 19)	P value
Age(y)	60.41 ± 7.75	63.0 ± 5.83	0.887
Sex (male)	26(50.98%)	16(84.21%)	0.012
Smoking history	20(39.21%)	15(78.95%)	0.03
Ki-67 (%)	27.97 ± 19.06	60.58 ± 18.86	< 0.001
Lesion location, n (%)	/	/	0.803
Right upper lobe	14(27.45%)	5(26.31%)	/
Right middle lobe	2(3.92%)	0(0.00%)	
Right lower lobe	12(23.53%)	3(15.79%)	
Left upper lobe	14(27.45%)	7(36.84%)	
Left lower lobe	9(17.65%)	4(21.05%)	

### 3.2 Comparison of IVIM and DKI histogram parameters between AC and SCC groups

**Figure 1:**
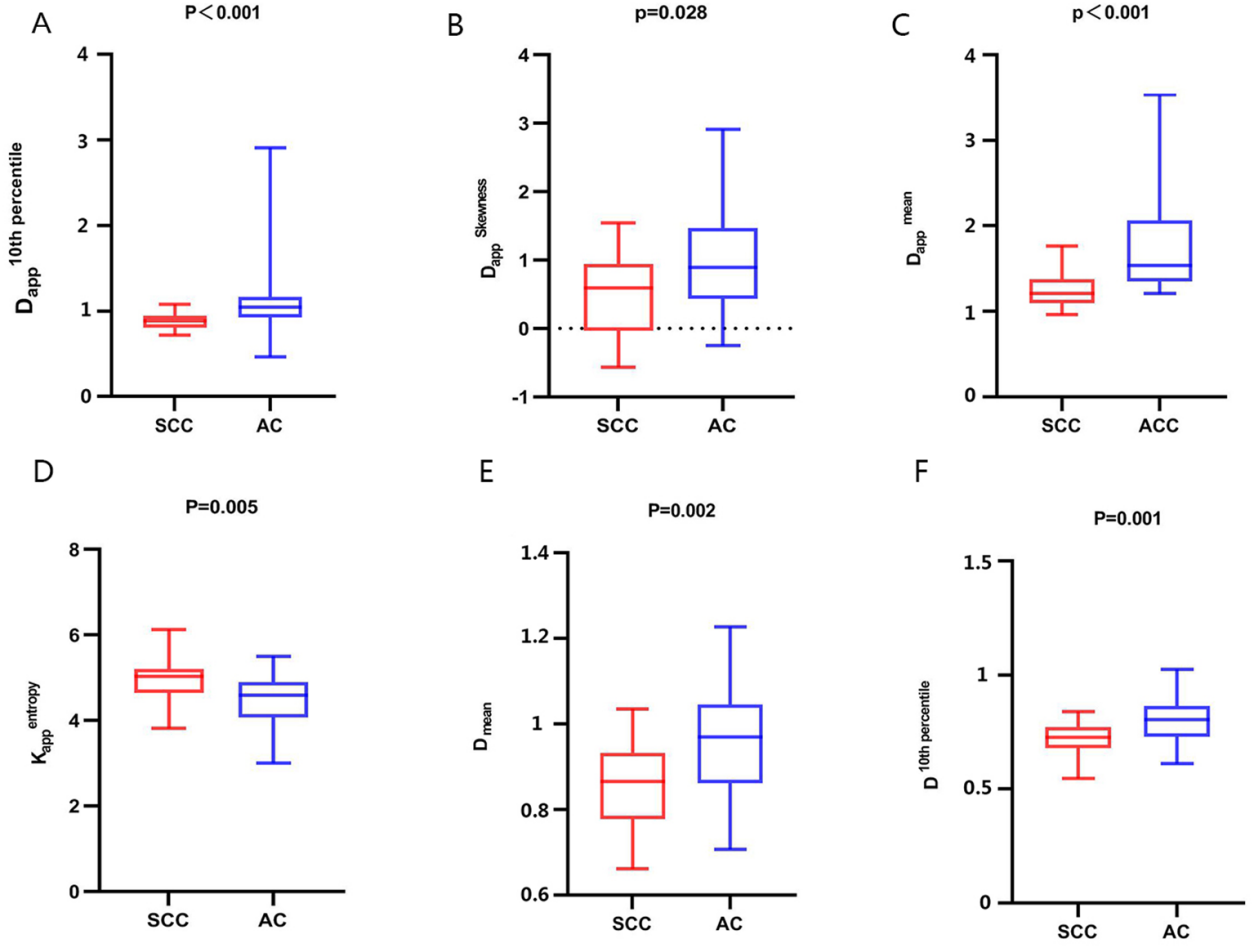
Comparison of whole-lesion histogram parameters of AC and SCC groups. **(A)**: D_app_^10th percentile^, **(B)**: D_app_^Skewness^, **(C)**: D_app_^mean^, **(D)**: K_app_^entropy^, **(E)**: D ^mean^, **(F)**: D ^10th percentile^.

**Figure 2:**
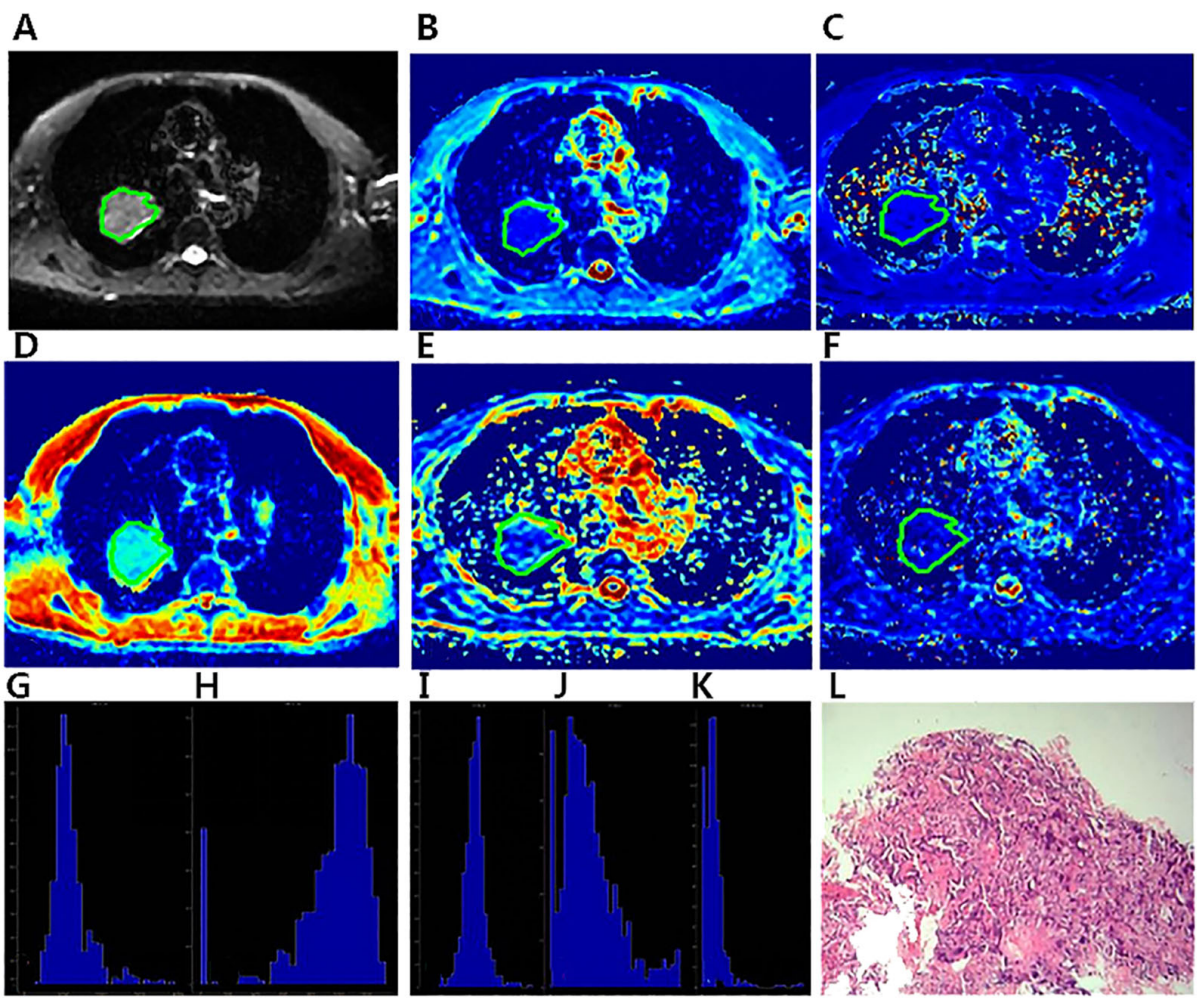
A 57-year-old male diagnosed with adenocarcinoma of the right Upper lobe.The ROI was delineated layer by layer on the original image **(A)** in the IVIM model, and then a VOI was synthesized and copied to the other functional pseudo-color images of D_app_ map **(B)**, K_app_ map **(C)**, D map **(D)**, f map **(E)** and D* map **(F)**. The whole- lesion histogram parameters of D_app_ **(G)**, K_app_ **(H)**, D **(I)**, f **(J)** and D* **(K)** value were calculated. Microscopic section from the poorly differentiated adenocarcinoma **(L)**.

**Figure 3:**
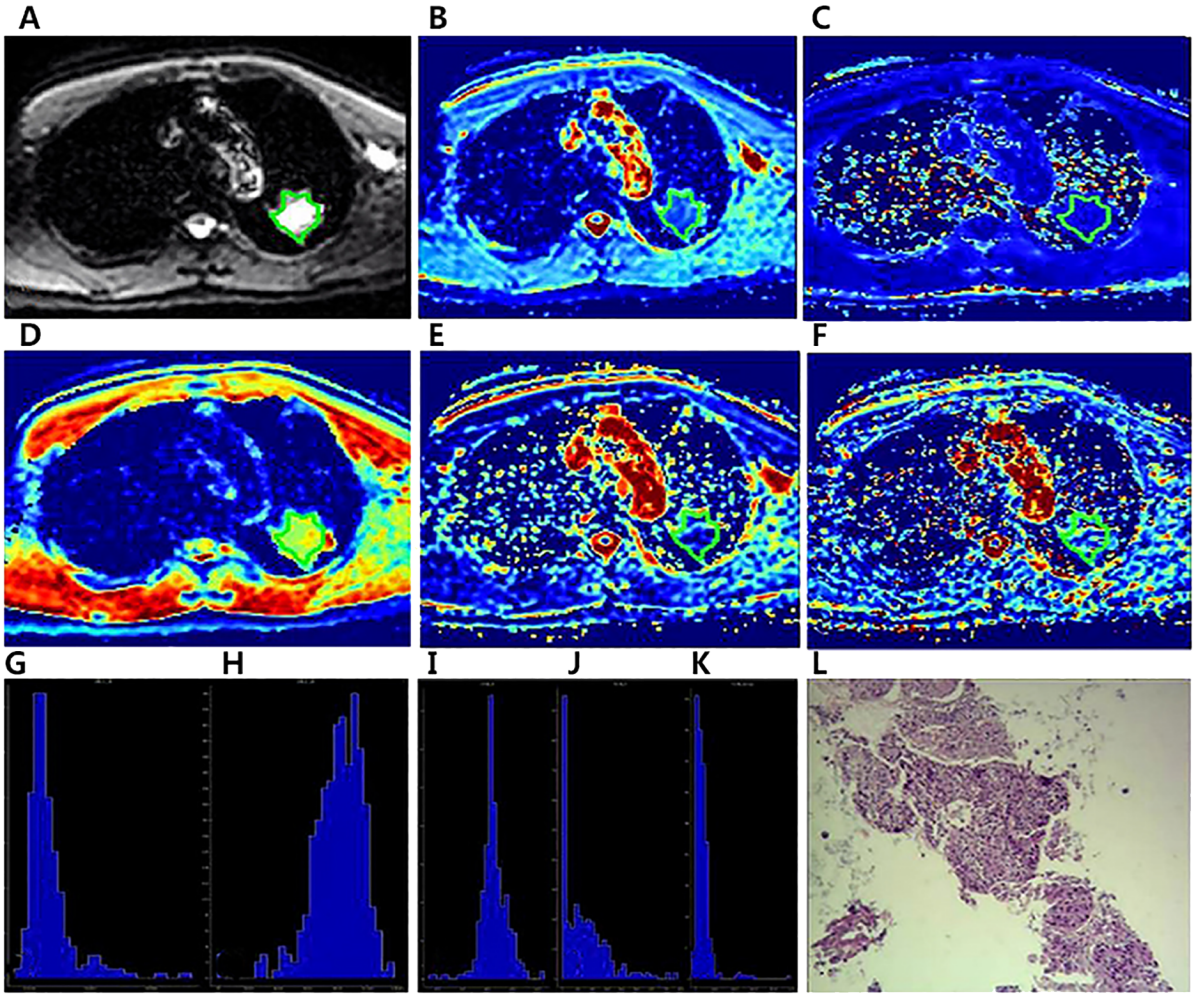
A 64-year-old male diagnosed with squamous cell carcinoma of the left Upper lobe.The ROI was delineated layer by layer on the original image **(A)** in the IVIM model, and then a VOI was synthesized and copied to the other functional pseudo-color images of D_app_ map **(B)**, K_app_ map **(C)**, D map **(D)**, f map **(E)** and D* map **(F)**.The whole-lesion histogram parameters of D_app_ **(G)**, K_app_ **(H)**, D **(I)**, f **(J)** and D* **(K)** value were calculated.Microscopic section from the poorly differentiated squamous cell carcinoma **(L)**.

The interobserver reproducibility of each histogram parameter for D, D*, f, K_app_, and D_app_ in the AC and SCC groups ranged from good to excellent. It was ultimately decided to utilize the mean values measured by two radiologists for statistical analysis. The D ^10th percentile^, D ^90th percentile^, D ^mean^, D ^median^, D_app_^10th percentile^, D_app_^90th percentile^, D_app_^mean^, D_app_^median^, D_app_^skewness^, and D_app_^SD^ of AC groups were significantly higher than those of the SCC groups, while the K_app_^entropy^, K_app_^SD^ of SCC groups were significantly higher than that of the AC groups. All the above differences were statistically significant (P < 0.05) (**Figure 1**, **Supplementary Table S1**). The differences in other histogram parameters between the AC and SCC groups were not statistically significant (P>0.05). The imaging features and whole-lesion histogram analysis of typical NSCLC (AC, SCC) are shown in **Figures 2**, **3** respectively.

### 3.3 Diagnostic efficacy of whole-lesion histogram parameters

**Figure 4:**
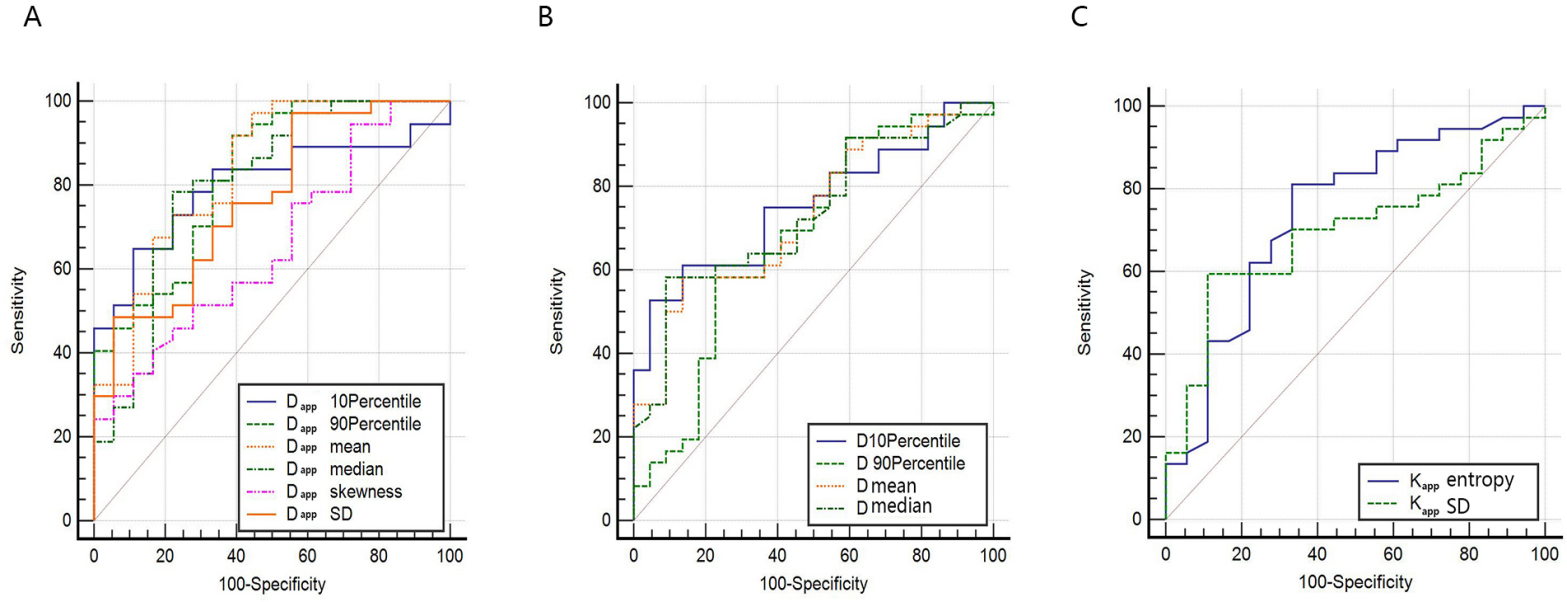
ROC curve for differentiating AC and SCC by whole-lesion histogram parameters. **(A)** ROC curves for histogram parameters of D_app_. **(B)** ROC curves for histogram parameters of D **(C)** ROC curves for histogram parameters of K_app_.

The ROC curves and the measurements of different histogram parameters are shown in **Figure 4** and **Table 3**, which are used to distinguish the AC and SCC groups. ROC curve analysis revealed that D_app_^mean^ showed the best performance for differentiating AC from SCC lesions, with an area under the ROC curve of 0.832 (95% confidence interval [CI]: 0.707-0.919).Using a D_app_^mean^ of 1.264 × 10^-3^ mm^2/^s as the optimal cut-off value, the sensitivity and specificity were 91.89%, 61.11%, respectively. Further pairwise comparisons of ROC curves did not yield significant differences in AUCs (all p > 0.05). D_app_^mean^ had the highest sensitivity (91.89%) and D_app_^skewness^ showed the highest specificity (100.00%) in differentiating AC and SCC lesions.

**Table 3:** ROC analysis results of histogram parameters for distinguishing AC and SCC groups.

Parameter	p value	AUC(CI95%)	Threshold	Sensitivity (%)	Specificity (%)
D^10th percentile^	<0.001	0.755(0.624-0.858)	>0.796	52.78	95.45
D^90th percentile^	0.019	0.678(0.542-0.795)	>1.076	58.33	77.27
D^mean^	<0.001	0.732(0.600-0.840)	>0.943	58.33	86.36
D^median^	<0.001	0.733(0.600-0.841)	>0.941	58.33	90.91
K_app_^entropy^	0.001	0.742(0.606-0.850)	≤4.921	81.08	66.67
K_app_SD	0.007	0.695(0.556-0.812)	≤0.224	59.46	88.89
D_app_10th percentile	<0.001	0.799(0.669-0.895)	>0.987	64.86	88.89
D_app_ 90thpercentile	<0.001	0.824(0.698-0.914)	>1.696	91.88	61.11
D_app_ mean	<0.001	0.832(0.707-0.919)	>1.264	91.89	61.11
D_app_^median^	<0.001	0.802(0.672-0.897)	>1.292	78.38	77.78
D_app_^skewness^	0.046	0.654(0.514-0.777)	>1.54	24.32	100
D_app_ SD	0.001	0.766(0.632-0.869)	>0.478	48.65	94.44

### 3.4 Correlation between histogram parameters and the Ki-67 index in NSCLC

**Figure 5:**
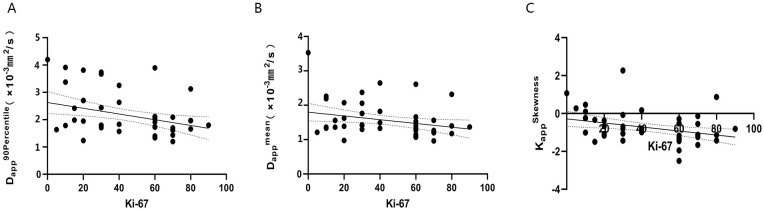
The correlation of D_app_^90thpercentile^, D_app_^mean^, K_app_^Skewness^ value and Ki-67 index in patients with NSCLC **(A–C)**. **(A)** r=-0.340, P<0.05; **(B)** r=-0.287, P<0.05; **(C)** r=-0.344, P<0.05.

DKI histogram parameters: D_app_^90thpercentile^, D_app_^mean^, K_app_^skewness^ showed a slight negative correlation with Ki-67 expression (r value -0.340, -0.287, -0.344, respectively; P< 0.05), while the other histogram parameters showed no significant correlation with Ki-67 expression(all P > 0.05). IVIM histogram parameters showed no significant correlation with Ki-67 expression(all P > 0.05), (**Figure 5**).

## 4 Discussion

Using whole-lesion volume histogram analysis, the present study suggests that multiple histogram parameters of IVIM (D) and DKI (D_app_, K_app_) can noninvasively and objectively evaluate the complexity and heterogeneity of the microenvironment structure in NSCLC. It is clinically feasible and has diagnostic value for AD and SCC. Furthermore, our study also indicates a certain correlation between the histogram parameter D_app_^90thpercentile^, D_app_^mean^, K_app_^skewness^ with the Ki-67 index, which provides a new parameter basis for evaluating the malignancy of NSCLC.

D_app_ and D are both diffusion-related parameters, where D represents pure molecular diffusion without the influence of microcirculation perfusion and D_app_ is the kurtosis corrected diffusion coefficient (13). Their values are mainly influenced by cell density and extracellular matrix components. Differences in D_app_ and D between AC and SCC have been inconsistent in previous studies using single-section ROI analyses. Some studies found no significant differences in D_app_ and D values between AC and SCC (16, 26), while others revealed significantly higher D_app_ or D values in AC compared to SCC (17, 27). Compared with previous studies on AC and SCC by DKI and IVIM, we used whole-lesion histogram analysis, which can more accurately reflect the degree of differences in the microenvironment structure of NSCLC subtypes. In this study, we found that compared to the SCC groups, the D ^10th percentile^, D ^90th percentile^, D ^mean^, D ^median^, D_app_^10th percentile^, D_app_^90th percentile^, D_app_^mean^, D_app_^median^, D_app_^skewness^ and D_app_^SD^ values in the AC groups were higher, while there was no significant difference in other histogram parameters of D_app_ and D between them.

In the analysis of whole lesion histograms, percentiles can reflect the distribution of voxels that form the histogram. The lower percentile of D_app_, D values in the histogram (such as the 10th percentile) may represent areas with higher cell density in the tumor; in contrast, higher percentiles (such as the 90th percentile) may reflect areas with lower cell density and lower diffusion limitations of water molecules (28). This result indicated that the degree of diffusion restriction of water molecules was significantly different between AC and SCC, both in the higher and lower tumor cell density regions, as well as in their diffusion mean and median values. The degree of diffusion restriction of water molecules in SCC was higher than that in AC. In terms of identification between SCC and AC, previous studies have shown that the cells were more densely packed in SCC than in AC (29). Therefore, the diffusion of water molecules in SCC is more restricted, and the results of this study are in general agreement with previous theories.

The histogram parameter SD reflects the degree of dispersion of the D_app_ voxel distribution within the entire lesion compared to the mean. The larger the value is, the greater the difference between the majority of values and the mean. Additionally, the skewness of the D_app_ reflects the heterogeneity of the voxel distribution in the histogram, The larger the skewness absolute value is, the greater the difference in diffusion within the lesion. In this study, the D_app_^SD^, D_app_^skewness^ of AC was higher than that of SCC. This indicates that there is a greater difference in the diffusion of water molecules per pixel in AC compared to SCC. It is speculated that the reason may be that tumor cells in AC tend to grow invasively along the outside of the tracheal or alveolar wall. In addition to adenocarcinoma tissues containing glandular lumens that store mucus, whereas tumor cells in squamous lung carcinomas exhibit predominantly clumped growth (30). This allows for greater variability in water molecule diffusion in different regions in AC. Therefore, compared to the average of single-section ROI analyses, which are greatly influenced by extreme values, histogram analysis can provide complex biological characteristics within the tumor and reflect the heterogeneity of the entire tumor.

D* and f are IVIM perfusion-related parameters. This study found no significant difference in the histogram parameters of D * or f values between AC and SCC. This indicates that the D * and f histogram parameters cannot reflect the characteristics of NSCLC well, and their differential diagnostic value for AC and SCC is relatively limited. In addition, previous studies revealed that measurement consistency of D* and f among different observers were relatively low (13, 15, 31), therefore the value of IVIM-related perfusion parameters needs to be further explored.

K_app_ is an important parameter of DKI, and its value is closely related to the complexity of the tumor tissue structure (32). In the histogram, entropy represents the complexity and nonuniformity of the image’s gray texture, while SD reflects the degree of dispersion of the image texture. In this study, the entropy, SD of the K_app_ value of SCC was significantly higher than that of AC, indicating that the tissue microenvironment structure was more heterogeneous and complex in SCC. Previous studies have shown that SCC has a shorter doubling time and faster proliferation rate than AC (33), while highly proliferative tumors have higher internal structural complexity due to increased cellularity, vascular proliferation, and necrosis (34). Our research results are consistent with previous research findings. Further ROC analysis indicated that the whole-lesion histogram analysis of IVIM and DKI has potential clinical value for the differentiation of NSCLC subtypes. Among them, D_app_^mean^ showed the best performance for differentiating AC from SCC lesions.

Ki-67 is a good indicator for evaluating tumor cell proliferation activity, and a higher Ki-67 index is associated with more active tumor cells and faster multiplication (35). In this study, the Ki-67 count of SCC was significantly higher than that of AC, consistent with a previous study (36). This suggests that the expression of Ki-67 in different pathological subtypes of NSCLC tissues varies, primarily due to the higher cell division rate of SCC compared to AC. Furthermore, our study explored the associations between IVIM and DKI histogram parameters and Ki-67 proliferation in NSCLC tumor tissues. We found that the histogram parameter D_app_^90th percentile^, D_app_^mean^, K_app_^skewness^ from DKI was slightly negatively correlated with Ki-67 expression. This may be due to the increase in tumor proliferative capacity (an increase in Ki-67 values), which leads to a denser number of cells inside the tumor and the proliferation of tumor blood vessels (35), making the diffusion more restricted and the internal structure more complex. In previous studies based on single section ROI analysis, Feng et al. demonstrated a weak negative correlation between D_app_ values and the Ki-67 index, while K_app_ values did not significantly correlate with Ki-67 expression in NSCLC (16). These findings align generally with our study. However, a study by Zheng et al. (16) for lung cancer suggested that the D value from IVIM was negatively correlated with Ki-67 expression, while the D_app_, D* and f values were not significantly correlated with Ki-67 expression in lung cancer. Discrepancies in findings may arise from differences in sample composition (including both SCC and AC in our study), variations in b-value parameters, and the use of whole-region histogram analysis in our study, which captures the overall heterogeneity within the tumor. In the future, it is necessary to expand the sample size and optimize scanning parameters for further research.

This study has several limitations. First, this study is a single center, small sample study with a relatively small number of cases, especially SCC cases. Therefore, it is necessary to further increase the sample size to achieve multicenter, large sample studies to explore and confirm the clinical application value of DKI and IVIM in NSCLC. Second, there is no unified standard for setting the b value. Although this study is based on the optimized b value discussed in multiple previous studies, deviations are inevitable, and a more comprehensive and standardized scanning plan needs to be developed in the future. Third, although larger areas of necrosis were excluded, smaller areas of necrosis might have been included due to manual delineation on the VOI, which could potentially affect the measurement results. Fourth, we did not evaluate the impact of tumor differentiation on differential diagnosis due to the limited number of cases. Future studies with larger sample sizes are needed to verify our findings.

## 5 Conclusion

In conclusion, our study demonstrates the value of IVIM and DKI histogram analyses in differentiating NSCLC subtypes, specifically AC and SCC, using non-invasive imaging techniques. The correlation between histogram parameters and the Ki-67 index provides valuable insights into tumor proliferation. Specifically, D_app_^mean^, D_app_^90th percentile^, and K_app_^skewness^ show potential as markers for assessing tumor aggressiveness. These findings support the use of IVIM and DKI as effective tools for NSCLC diagnosis and treatment planning.

## Data Availability

The original contributions presented in the study are included in the article/**Supplementary Material**. Further inquiries can be directed to the corresponding authors.
