# Supplementary material for: The value of multiple diffusion metrics based on whole-lesion histogram analysis in evaluating the subtypes and proliferation status of non-small cell lung cancer
Source: Front Oncol. 2024 Oct 30;14:1434326. doi: 10.3389/fonc.2024.1434326 (PMC11557419; doi:10.3389/fonc.2024.1434326)
Supplement: Supplementary file 1 [file DataSheet1.docx]

Supplementary Material

**TABLE** **S1 Comparison of different histogram parameters between the AC and SCC groups****（×10^-3^㎜^2^/s）.**

| Parameter | AC(n=51) | SCC (n= 19) | p value |
| --- | --- | --- | --- |
| **D *histogram*** | | | |
| 10th percentile | 0.81 ± 0.10 | 0.71 ± 0.08 | 0.001 |
| 90th percentile | 1.10(0.97,1.18) | 0.99(0.87,1.08) | 0.024 |
| Mean | 0.95 ± 0.12 | 0.85 ± 0.10 | 0.002 |
| Median | 0.95 ± 0.14 | 0.85 ± 0.11 | 0.003 |
| Entropy | 4.81(4.38,5.13) | 4.49(4.22,4.87) | 0.079 |
| Uniformity | 0.04(0.03,0.06) | 0.06(0.04,0.07) | 0.061 |
| Kurtosis | 3.54(2.63,4.43) | 3.98(3.27,4.71) | 0.332 |
| Skewness | -0.26(-0.57, -0.38) | -0.18(-0.49,0.03) | 0.432 |
| SD | 0.20(0.14,0.27) | 0.16(0.12,0.20) | 0.061 |
| ***D* histogram*** | | | |
| 10th percentile | 0.71(0.56,0.81) | 0.70(0.68,0.76) | 0.634 |
| 90th percentile | 1.56(1.06,1.88) | 1.41(1.26,1.58) | 0.593 |
| mean | 0.72(0.53,0.95) | 0.67(0.59,0.82) | 0.593 |
| Median | 0.52(0.39,0.66) | 0.51(0.42,0.60) | 0.848 |
| Entropy | 2.48(1.25,5.22) | 4.68(1.79,7.27) | 0.161 |
| Uniformity | 0.07(0.04,0.10) | 0.05(0.03,0.08) | 0.358 |
| Kurtosis | 2.99(1.24,1.12) | 7.51(3.43,1.34) | 0.146 |
| Skewness | 1.75(1.09,3.26) | 2.59(1.23,6.47) | 0.276 |
| SD | 0.88(0.64,1.26) | 0.90(0.56,1.07) | 0.738 |
| ***f histogram*** | | | |
| 10th percentile | 0.00(0.00,0.04) | 0.00(0.00,0.00) | 0.560 |
| 90th percentile | 0.35 ± 0.19 | 0.33 ± 0.11 | 0.080 |
| mean | 0.18(0.11,0.26) | 0.15(0.12,0.18) | 0.202 |
| Median | 0.13(0.08,0.26) | 0.14(0.09,0.17) | 0.514 |
| Entropy | 3.90(3.33,4.41) | 3.85(3.33,4.14) | 0.536 |
| Uniformity | 0.11(0.07,0.18) | 0.11(0.07,0.14) | 0.640 |
| Kurtosis | 2.69(2.07,3.94) | 3.09(2.52,3.86) | 0.165 |
| Skewness | 0.81(0.22,1.14) | 0.83(0.57,1.05) | 0.920 |
| SD | 0.16(0.10,0.18) | 0.12(0.09,0.19) | 0.172 |
| **K_app_ histogram** | | | |
| 10th percentile | 0.35(0.00,0.57) | 0.18(0.00,0.49) | 0.318 |
| 90th percentile | 1.06(0.88,1.27) | 1.07(0.97,1.25) | 0.111 |
| Mean | 0.74(0.63,0.91) | 0.78(0.67,0.91) | 0.929 |
| Median | 0.82 ± 0.24 | 0.89 ± 0.19 | 0.682 |
| Entropy | 4.45 ± 0.57 | 4.91 ± 0.54 | 0.005 |
| Uniformity | 0.06(0.04,0.08) | 0.04(0.03,0.05) | 0.021 |
| Kurtosis | 3.97(2.23,5.03) | 3.15(2.40,4.59) | 0.157 |
| Skewness | -0.64(-1.13, -0.26) | -0.77(-1.13, -0.46) | 0.107 |
| SD | 0.21(0.18,0.31) | 0.30(0.26,0.33) | 0.020 |
| **D_app_** **histogram** | | | |
| 10th percentile | 0.91 ± 0.20 | 0.90 ± 0.41 | P<0.001 |
| 90th percentile | 2.41(1.81,3.32) | 1.87(1.64,2.21) | P<0.001 |
| Mean | 1.53(1.28,1.96) | 1.37(1.22,1.96) | P<0.001 |
| Median | 1.40(1.15,1.69) | 1.27(1.18,1.59) | P<0.001 |
| Entropy | 5.84(5.23,6.18) | 5.76(5.52,6.10) | 0.405 |
| Uniformity | 0.02(0.02,0.03) | 0.02(0.02,0.03) | 0.577 |
| Kurtosis | 4.83(3.00,9.05) | 5.27(4.10,12.02) | 0.523 |
| Skewness | 0.95 ± 0.76 | 0.48 ± 0.60 | 0.028 |
| SD | 0.45(0.29,0.78) | 0.33(0.21,0.44) | 0.001 |

**
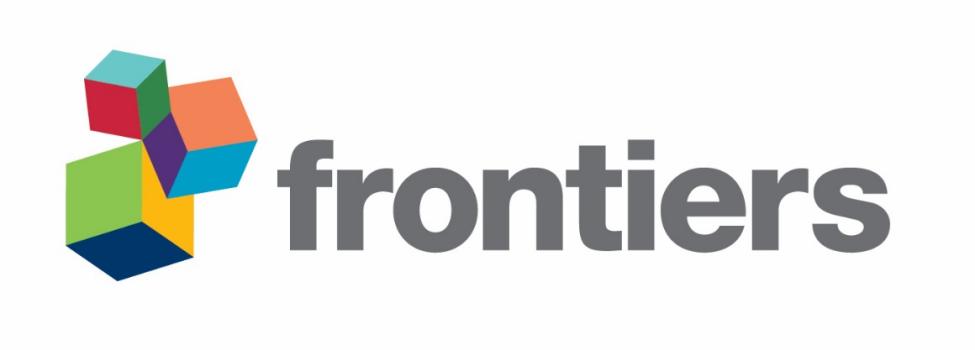
**

**Supplementary Figure 1.** The figure legends are required to have the same font as the main text, 12 point normal Times New Roman, single spaced. Please use a single paragraph for each legend and prepare the figures keeping in mind the PDF layout.
