# Supplementary figures and images for: The value of multiple diffusion metrics based on whole-lesion histogram analysis in evaluating the subtypes and proliferation status of non-small cell lung cancer
Source: Front Oncol. 2024 Oct 30;14:1434326. doi: 10.3389/fonc.2024.1434326 (PMC11557419; doi:10.3389/fonc.2024.1434326)

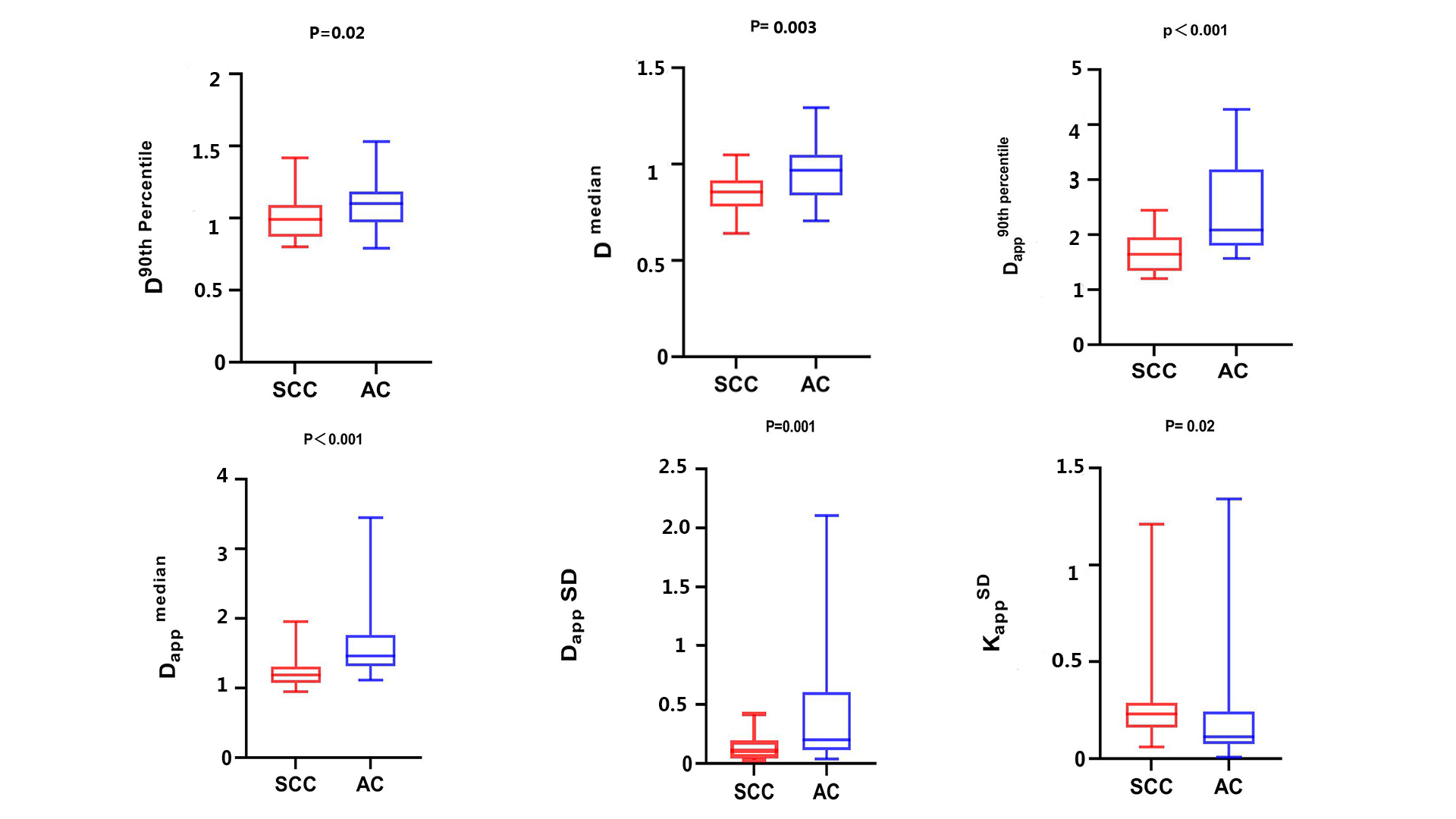

Supplement: Supplementary file 2 [file Image1.jpg]
